# Unintended dural tears during unilateral biportal endoscopic lumbar surgery: incidence and risk factors

**DOI:** 10.1007/s00701-024-05965-8

**Published:** 2024-02-21

**Authors:** Hang Yu, Qingzhong Zhao, Jianwei Lv, Jianjun Liu, Bin Zhu, Lei Chen, Juehua Jing, Dasheng Tian

**Affiliations:** 1 Department of Orthopaedics & Spine Surgery, The Second Hospital of Anhui Medical University, No.678 Furong Road, Economic and Technological Development Zone, Hefei, 230601 China; 2 Department of Orthopaedics, Huzhou Central Hospital, 313000 Huzhou, China

**Keywords:** Dural tear, Endoscopic spinal surgery, Lumbar spinal stenosis, Revision surgery, Risk factors, Unilateral biportal endoscopic surgery

## Abstract

**Background:**

An unintended dural tear (DT) is the most common intraoperative complication of lumbar spine surgery. The unilateral biportal endoscopic technique (UBE) has become increasingly popular for treating various degenerative diseases of the lumbar spine; however, the DT incidence and risk factors specific to UBE remain undetermined. Therefore, this study aimed to evaluate the incidence and risk factors of DTs in UBE.

**Method:**

Data from all patients who underwent UBE for degenerative lumbar spinal diseases from November 2018 to December 2021 at our institution were used to assess the effects of demographics, diagnosis, and type of surgery on unintended DT risk.

**Results:**

Overall, 24/608 patients (3.95%) experienced DTs and were treated with primary suture repair or bed rest. Although several patients experienced mild symptoms of cerebrospinal fluid (CSF) leaks, no serious postoperative sequelae such as nerve root entrapment, meningitis, or intracranial hemorrhage occurred. Additionally, no significant correlations were identified between DT and sex (*P* = 0.882), body mass index (BMI) (*P* = 0.758), smoking status (*P* = 0.506), diabetes (*P* = 0.672), hypertension (*P* = 0.187), or surgeon experience (*P* = 0.442). However, older patients were more likely to experience DT than younger patients (*P* = 0.034), and patients with lumbar spinal stenosis (LSS) were more likely to experience DT than patients with lumbar disc herniation (LDH) (*P* = 0.035). Additionally, DT was more common in revision versus primary surgery (*P* < 0.0001) and in unilateral laminotomy with bilateral decompression (ULBD) versus unilateral decompression (*P* = 0.031). Univariate logistic regression analysis revealed that age, LSS, ULBD, and revision surgery were significant risk factors for DT.

**Conclusions:**

In this UBE cohort, we found that the incidence of DT was 3.95%. Additionally, older age, LSS, ULBD, and revision surgery significantly increased the risk of DT in UBE surgery.

## Introduction

Unintended dural tears (DTs) are frequent complications of lumbar spinal surgery. In previous studies, the incidence of DT has varied greatly and depended on various factors, including demographic characteristics, diagnosis, surgical history (primary versus revision), and invasiveness [2, 3, 5, 11, 13, 18, 19, 24, 26, 27, 30, 31, 33, 37–39]. Inappropriate tear management may result in persistent cerebrospinal fluid (CSF) leaks and the formation of pseudomeningoceles or cutaneous fistulas [3, 8], which, in turn, leads to symptoms of low CSF pressure such as headache and nausea. It is also possible for patients to experience more severe complications, including nerve root entrapment [21, 22], meningitis [16, 34], surgical site infections [1, 8], and intracranial hemorrhage [7, 17, 40]. In summary, the effects of DTs may result in increased healthcare costs and poor patient satisfaction [20, 25, 35].

In 2013, Soliman [28] used the unilateral biportal endoscopic (UBE) technique to treat lumbar disc herniation (LDH) according to the first technical description by Antoni and Claro from 1996 [4]. With the continuous optimization of surgical instruments and improvement of surgical techniques, UBE has gradually been implemented to treat various spinal degenerative diseases, including LDH, lumbar spinal stenosis (LSS) [10, 29], degenerative lumbar spondylolisthesis (DLS) [9, 14], and cervical radiculopathy [12, 23]. Compared with open surgery, UBE possesses the general merits of minimally invasive surgery, including less trauma, less blood loss, and faster recovery [15]. Furthermore, this technique is better than single-portal spinal endoscopic surgery because of the division, and thus, lack of interference, between its two percutaneous portals. Surgical instruments and endoscopes can therefore be moved freely without portal limitations, enabling convenient and flexible operations.

Although many previous studies have already reported the incidence of DTs associated with minimally invasive spine surgery, literature on DTs resulting from UBE is scarce [24]. Therefore, this study aimed to evaluate the incidence and risk factors of DTs in UBE.

## Methods and materials

### Patient population

We retrospectively evaluated the data of patients who experienced DTs from an initial cohort of 608 consecutive patients who had undergone UBE for degenerative lumbar spine disorders between November 2018 and December 2021 at our institution.

The inclusion criteria were as follows: (1) neurogenic claudication or radicular leg pain due to degenerative lumbar spine disorders that persisted longer than 6–8 weeks despite conservative treatment; (2) computed tomography and magnetic resonance imaging confirmation of a degenerative lumbar spine disorder including lumbar disc herniation, lumbar spinal stenosis, degenerative spondylolisthesis and spondylolisthesis; (3) UBE primary surgery performed to treat the degenerative lumbar spine disorder including the single-segment surgery and multi-segment surgery; (4) UBE revision surgery performed to treat the recidivate hernias and restenosis.

The exclusion criteria were as follows: (1) aged < 18 years; (2) concomitant issues such as traumatic injuries, primary infections, and tumors; and (3) other types of minimally invasive spine procedures, such as microendoscopic lumbar decompressive surgery, and percutaneous transforaminal endoscopic discectomy.

The study was approved by our institutional review board and informed consent was obtained from all patients (sl⁃xjs2019⁃001).

### Surgical procedure

#### Patient preparation

All surgeries were performed under general endotracheal anesthesia. The patient was placed prone on a radiolucent frame with an H-shaped pillow placed underneath. Thus, the abdomen was suspended and increased abdominal pressure was avoided.

#### Skin incisions and making portals

Following target level confirmation under C-arm fluoroscopic guidance, the radiolucent frame was adjusted so that the operative intervertebral disc space was perpendicular to the floor. Two incision markers were placed at the inner margin of the pedicle, 1 cm above and below the midline of the intervertebral space. The skin and subcutaneous tissues were pierced perpendicularly according to the incision markings, and serial dilators were inserted into the two incisions to dissect the muscle and form two portals. For a left-sided approach, the left-hand portal was approximately 6 mm in diameter and used as the observation portal for endoscope placement, while the right portal was approximately 10 mm in diameter and was employed as the working portal to manipulate surgical instruments.

#### Creating an extraforaminal working space

Once the endoscope was inserted into the observation portal, a saline irrigation system was used to keep the operative field clean. The system was gravity-driven and hung 50–60 cm above the patient. The soft tissues covering the operative intervertebral disc space were dissected using a radiofrequency knife to create an initial extraforaminal working space.

#### Laminotomy and ligamentum flavum removal

When the inner edge of the facet joint, lower edge of the superior lamina, upper edge of the inferior lamina, and superficial layer of the ligamentum flavum were exposed, an endoscopic drill and Kerrison punches were used to perform laminotomy to expose the superior margin of the ligamentum flavum. The ligamentum flavum and dural sac were detached carefully using a curette. The ligamentum flavum was then peeled from the cranial end down to the caudal end using a Kerrison punch to expose the dural sac and traversing nerve root.

#### Decompression

After flavectomy, the nerve root adjacent to the dural sac was exposed, and a Kerrison punch and drills were used to enlarge the working space as needed.

Discectomy was required for cases of symptomatic LDH. The surgeon used pituitary forceps to remove the herniated disc, while the dural sac and nerve root were protected by an assistant using a retractor. If lateral recess decompression was required, the operator preferred medial facetectomy to decompress the transverse nerve root.

For patients with DLS requiring interbody fusion, we preferred to perform biportal endoscopic transforaminal lumbar interbody fusion (BETLIF). An endoscopic drill and a Kerrison punch were used to perform ipsilateral laminectomy, contralateral sublaminar decompression, and flavectomy. After the unilateral traversing root was completely exposed, a Kerrison punch was used to perform unilateral facetectomy, and the disc was removed using pituitary forceps. Curettes were then used to remove the cartilaginous endplate and expose the subchondral bone. Once the endplate preparation was sufficient under endoscopic visualization, autologous bone debris chips from the lamina and facet were introduced into the disc space, using a specialized cannula.

Finally, a cage packed with autologous bone was inserted under fluoroscopic guidance, with the nerve roots retracted by the operative assistant. Two ipsilateral pedicle screws were inserted into the two portals, and two contralateral pedicle screws were inserted into two new contralateral incisions.

#### Wound closure

After sufficient decompression and meticulous hemostasis, the endoscope and instruments were removed. Subsequently, a small drainage catheter was inserted through the working portal to prevent the epidural hematoma, followed by wound closure with two single stitches.

### Unintended DTs

Unintended DTs were defined as the disruption of dural integrity with or without CSF leakage. If the arachnoid mater was visible during surgery, a DT was considered present, regardless of the presence of CSF leaks. CSF leakage was defined as the escape of CSF from the skin incision. The occurrence of unintended DTs was indicated by a presentation of low CSF pressure, including headache and nausea, after substantial postoperative drainage volume.

Patients with DTs measuring less than 10 mm were treated with bed rest and inpatient observation for 3–5 days, while primary suture repair was attempted for tears measuring more than 10 mm.

### Study measures

The following four categories of potential factors were examined: (1) the patients’ demographic characteristics, including age, sex, body mass index (BMI), smoking, diabetes, and hypertension; (2) preoperative diagnosis, e.g., LDH, LSS, and DLS; (3) revision versus primary surgery; and (4) surgical type, including unilateral decompression (UD), unilateral laminotomy for bilateral decompression (ULBD), and transforaminal lumbar interbody fusion using the UBE technique (BETLIF). Following the surgery, the surgeon documented the occurrence and details of the DT, as well as management or repair techniques.

BMI was defined based on the BMI classification of Asian people by WHO and that of Chinese people by the Chinese Medical Association [36]. Accordingly, weight was classified as normal when the BMI was < 24, overweight when the BMI was > 24, and obese if the BMI was > 28.

### Statistical analysis

The relationship between DTs and potential risk factors was subsequently analyzed. Pearson’s chi-square test was used to evaluate categorical differences between the two groups, while univariate logistic regression analyses were used to calculate the relative risk (RR) and 95% confidence intervals (CI). All statistical analyses were performed using SPSS Statistics version 26 (IBM Corporation, Armonk, NY, USA), and statistical significance was determined at a *P* value of 0.05.

## Results

Demographic characteristics of the patients are provided in Table 1. Between November 2018 and December 2021, 608 consecutive patients (295 women, 313 men) underwent UBE. The mean patient age was 54 years, and LDH was the most common preoperative diagnosis (385 cases), followed by LSS (144 cases) and DLS (71 cases).

**Table 1:** Summary of patient characteristics

Mean age (y) at surgery (range)		53.90（18-87）
	＜65	444
	≥65	164
Gender		
	Male	313
	Female	295
BMI		
	BMI＜24	274
	24≤BMI＜28	248
	28≤BMI	86
Diabetics		72
Hypertension		180
Smoker		143
Diagnosis		
	Lumbar disc herniation	385
	Lumbar spinal stenosis	144
	Degenerative spondylolisthesis	71
	Spondylolysis	8
Surgical segments		
	Single-segment surgery	500
	Multi-segment surgery	108
Surgery type		
	UD	408
	ULBD	120
	BETLIF	80
Revision surgery		14

**Fig. 1:**
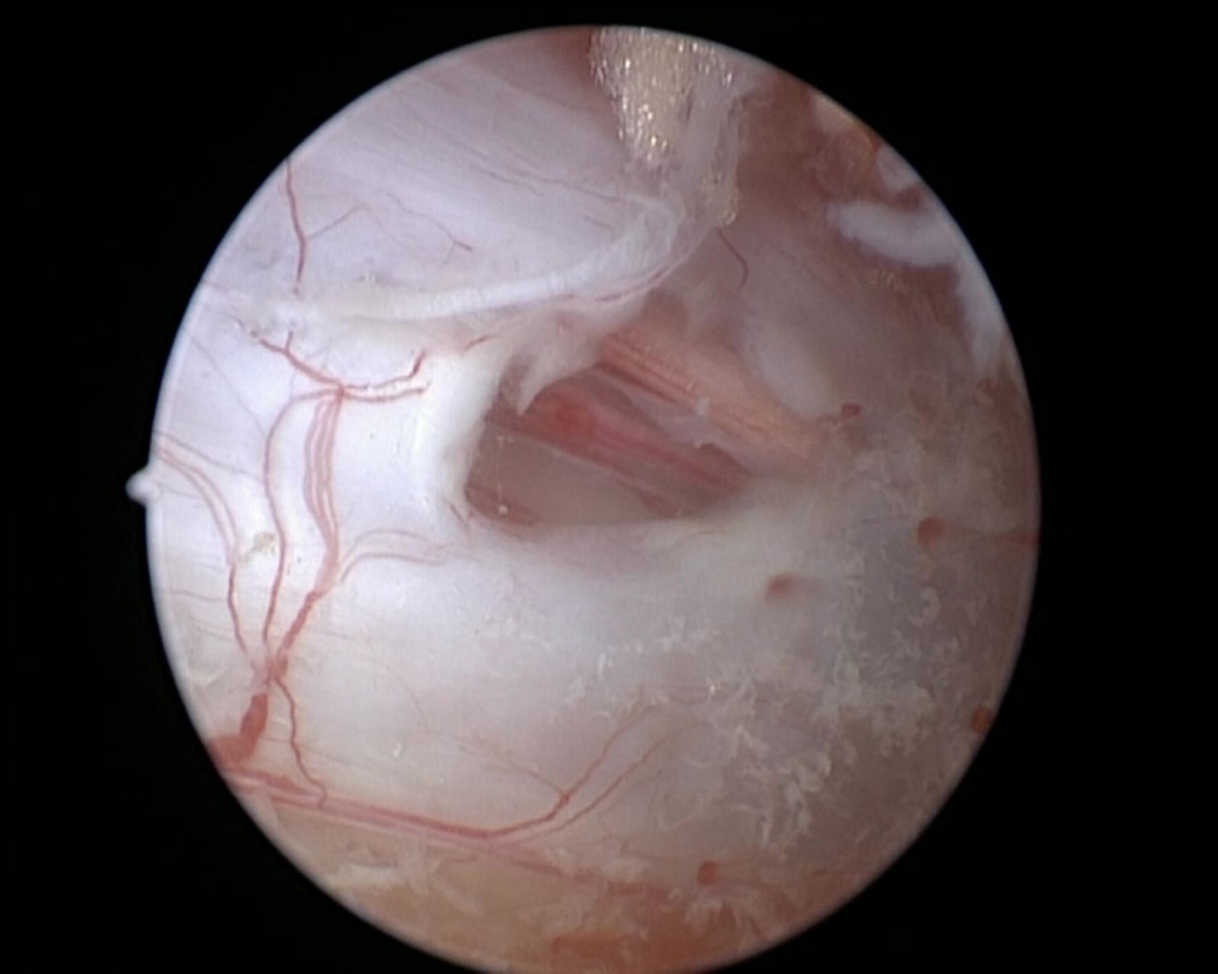
Dural tear during UBE surgery

The incidence of unintended DTs is reported in Table 2. DTs were observed in 24 patients (ten women, 12 men; 3.95%). Compared to patients without DTs, those with DTs were significantly older (mean age, 61 years); however, sex distribution did not differ between patients with and without DT. In addition, BMI, smoking status, and diabetes did not significantly increase the risk of DTs during UBE surgery (Fig. 1). In this study, patients with LSS and DLS were more likely to experience DTs, with rates of 6.85% and 4.35%, respectively, compared to patients with LDH (2.86%). Furthermore, unintended DTs occurred in 2.93% and 6.72% of patients who had undergone bilateral decompression and UD, respectively, and unintended DTs were significantly more common in patients who underwent revision surgery (28.57%) than in those who underwent primary surgery (3.37%).

**Table 2:** Incidence of dural tears according to demographics, diagnosis, surgery type, revision surgery and surgeon’s experience

	No. of DTs	Total no. of cases	Incidence (%)	P
Total No. of patients	24	608	3.95	
Age (y), mean (SD)	61.21			
Age:＜65 years	13	444	2.93	0.034
Age:≥65 years	11	164	6.71	
Gender				
Male	12	313	3.83	0.882
Female	12	295	4.07	
BMI				
Normal weight	9	274	2.94	0.758
Overweight	11	248	4.19	
Obese	4	86	4.00	
Smoking				
Yes	7	143	4.90	0.506
No	17	465	3.66	
Diabetes				
Yes	4	72	5.56	0.672
No	20	536	3.73	
Hypertension				
Yes	10	180	5.56	0.187
No	14	428	3.27	
Diagnosis				
Lumbar disc herniation	11	385	2.86%	
Lumbar spinal stenosis	10	146	6.85%	0.035
Degenerative spondylolisthesis	3	69	4.35%	0.778
Spondylolysis	0	8	-	-
Surgery type				
UD	11	408	2.93%	
ULBD	9	120	6.72%	0.031
BETLIF	4	80	5.00%	0.461
LSS-UD	2	73	2.74%	
LSS-ULBD	8	71	11.27%	0.092
Revision surgery				
Yes	4	14	28.57%	＜0.0001
No	20	594	3.37%	
Surgical experience				
First 100 patients with LDH	5	95	5.00	0.442
Last 100 patients with LDH	2	98	2.00	

LSS-UD mean patients with LSS underwent UD

LSS-ULBD mean patients with LSS underwent ULBD

All procedures were performed by a senior spine surgeon with extensive experience who received UBE-associated training at several spinal centers. To investigate the influence of the surgeon’s experience on the risk of DT, we analyzed the incidence of dural tears in the first 100 patients and last 100 patients separately. Moreover, to avoid the influence of other factors, 200 patients who underwent primary UBE for LDH treatment were also analyzed. The incidence of tears was 5% among the first 100 LDH patients and 2% among the last 100 LDH patients. However, the difference in tear incidence between the groups was not significant (*P* = 0.465).

An overview of the univariate analysis of DT risk factors is presented in Table 3. The results indicated that age (odds ratio [OR] = 2.384, *P* = 0.039), LSS (OR = 2.500, *P* = 0.041), ULBD (OR = 2.224, *P* = 0.088), and revision surgery (OR = 11.480, *P* < 0.0001) were significant risk factors. Additionally, unintended DTs were recognized during surgery in 18/24 patients, and postoperatively in the remaining six. Only one of the 18 patients whose DT was identified during the primary surgery received suturing during surgery, and the remaining 23 patients received careful observation. Of the 24 patients, eight experienced headaches and nausea due to low CSF pressure, which recovered gradually within a few days with bed rest. Furthermore, one patient developed postoperative delirium, which may have been caused by spinal cord hypertension due to intraoperative saline irrigation; however, no wound infections or subcutaneous fluid collections occurred, and revision surgery was not required.

**Table 3:** Univariate logistic regression analysis results for the risk of dural tears

	OR	95% CI	P
Age (y), mean (SD)			
Age:＜65 years	1	-	-
Age:≥65 years	2.384	1.046-5.433	0.039
Diagnosis			
Lumbar disc herniation	1	-	-
Lumbar spinal stenosis	2.5	1.038-6.019	0.041
Degenerative spondylolisthesis	1.545	0.420-5.689	0.513
Surgery type			
UD	1	-	-
ULBD	2.224	0.888-5.568	0.020
ULIF	1.654	0.520-5.260	0.284
LSS-UD	1		
LSS-ULBD	4.508	0.923-22.022	0.063
Revision surgery			
Yes	11.480	3.315-39.761	＜0.0001
No	1	-	

LSS-UD mean patients with LSS underwent UD

LSS-ULBD mean patients with LSS underwent ULBD

## Discussion

An unintended DT is a common intraoperative complication of degenerative lumbar spine surgery, with an incidence ranging from 1.6 to 15.8% [2, 3, 5, 8, 11, 13, 18, 19, 24, 26, 27, 30–33, 37–39]. In this current study, 3.95% of patients undergoing UBE experienced DT, and this value is consistent with those from previous studies. Several factors affect the incidence of DT, including diagnosis and surgical history (revision versus primary), as well as surgical type.

Preoperative diagnosis determines the surgical goals and surgical type selected, thus affecting the degree of surgical invasiveness and complexity, which, in turn, impacts the risk of DTs [2]. Previously, Smorgick et al. [27] conducted a prospective study of 523 patients undergoing spine surgery and found DT rates of 4.6% for LDH, 12.8% for LSS, and 4.18% for DLS. Furthermore, Du et al. [5] reviewed data from 4822 patients and found that the diagnosis of LSS was a significant preoperative risk factor. Our study numbers were comparable to those of these previous studies, and DT was more common in patients with LSS than in patients with LDH.

Compared to LDH patients, patients with LSS presented with more severe degenerative changes in the lumbar spine, including hypertrophy of the laminae, facet joints, and ligamentum flavum, as well as instability of lumbar spine. These degenerative changes may account for the adherence of the joint capsule and ligamentum flavum to the dura mater, increasing the risk of DTs when the facet joints and ligamentum flavum are removed. Patients with LSS have severe degenerative changes and often require multi-segmental or bilateral decompression to completely decompress and relieve symptoms. This increases the invasiveness and complexity of the required procedure and prolongs operative time. Thus, the likelihood of DT occurrence also increases. Additionally, patients with LSS tend to be older than those with LDH. As a result, the dura mater is more fragile and is more easily torn.

BETLIF would seem more likely to cause DTs due to its greater surgical invasiveness and technical demands. However, many previous studies have shown that the incidence of DTs among patients who underwent laminectomy with instrumented fusion appears to be comparable to that seen among patients who underwent laminectomy alone [5, 27, 38]. This outcome is similar to our results. However, we were unable to identify a significant difference between the incidence of DT related to laminectomy without instrumented fusion and DT incidence related to BETLIF. Compared with laminectomy without instrumented fusion, the BETLIF procedure requires the exposure of a broader surgical field. This improves visualization and reduces the DT risk. Moreover, postoperative drainage volumes are increased in BETLIF, which can obfuscate the presence of CSF leaks and result in failure in identification of DTs.

Previously, Tsutsumimoto [33] collected prospective data from 555 patients and found DT rates of 3.78% for UD and 12.5% for ULBD. Similarly, a higher DT rate in patients who underwent ULBD was also observed in our current study. However, most patients underwent ULBD for relief of neurogenic claudication symptoms due to LSS, which is considered a risk factor for DT, as previously described. To exclude the influence of preoperative diagnosis on DTs, we analyzed the incidence of DTs in patients with LSS who underwent ULBD and UD and found no significant difference between the two groups. Therefore, it appears that LSS is the reason for the high incidence of DTs in ULBD. In our study, out of 73 patients with LSS who underwent ULBD, only two patients experienced DTs. However, any conclusion drawn from this small sample size is unreliable and requires further study with larger sample size.

A significantly increased DT risk has been reported with revision procedures [2, 5, 11, 27, 30–32, 38]. In a study of 1549 cases, Tafazal and Sell [32] found DT rates of 3.5% and 13.2% in primary and revision discectomy, respectively. Additionally, using multivariate analysis of detailed data from 4652 patients, Ishikura et al. [11] identified revision surgery as a vital risk factor for DTs. In our study, we also found that unintentional DTs were significantly more common among patients who had previously undergone surgery. Therefore, revision surgery may cause scar formation at the surgical site, leading to dural adhesion to adjacent tissue and thus affecting the recognition of normal tissue structure, which may be responsible for the high incidence of DTs in revision surgery.

Aging is also known to adversely affect outcomes in spinal surgery. Challenges related to this include poor surgical wound healing, surgical site infections, failure to fusion, and more reoperations. Numerous studies have demonstrated that unintended DT is common in older patients [2, 5, 11, 26, 27, 31, 33, 38, 39]. In fact, one previous study found that patients with DTs were older than those without [33]. Therefore, there is no doubt that when the patient group is more advanced in age, LSS, and DLS predominate. In contrast, LDH is more prevalent in younger patients. Pathological variations alter the goal of surgery and procedure selection, and these variables may affect DT incidence.

Strömqvist et al. [31] classified 64,431 patients into three diagnostic groups: LDH, LSS, and DLS. They found that older patients were more likely to experience DTs, regardless of the diagnosis [31]. This is due to the fragile dura mater that occurs with aging, as well as adhesions between the dura sac and surrounding tissue. Comparably, in the current study, we found that patients with DTs were significantly older than those without DTs.

In addition to aging, preoperative diagnosis, and surgical type, other variables such as concomitant chronic diseases (diabetes, hypertension, or obesity) and harmful habits like smoking may also influence the occurrence of DTs during spinal surgery. However, none of these factors significantly affected DT risk in our study.

Regarding the surgeon’s surgical experience, it remains controversial whether it affects the incidence of DTs. Some studies suggest that surgical experience does not significantly affect the incidence of DTs [18, 19]; however, other studies have reported that less experienced surgeons cause more DTs [26, 33, 37]. In this current study, we did not identify any significant correlations between the incidence of DTs and the experience of the surgeon.

Primary suture repair is the gold standard for the management of intraoperative DTs [6]. However, UBE offers limited working space; therefore, suturing the dura can be challenging. Additionally, the decreased operative space in UBE reduces the risks of persistent CSF leak and pseudo-meningocele formation. We found that most DTs did not require primary suture repair and healed spontaneously. We believe that if the dural tear was identified during the operation, the operation should be terminated as soon as possible to avoid adverse consequences such as intracranial hypertension caused by long-term intradural water pressure perfusion. Dural tears < 10 mm in size are usually treated with 72 h of bed rest and in-hospital observation while those > 10 mm in size need to be sutured. Although a few patients experienced headaches and nausea, the symptoms of low CSF pressure were relieved with a few days of bed rest. Moreover, we did not observe any serious sequelae in patients who were conservatively managed.

This study had several limitations. First, as a single-center study, selection bias may have occurred during patient enrollment and resulted in either overestimations or underestimations. Second, since most data points were categorical rather than continuous, potential covariates could not be assessed. For example, smoking history was recorded as “yes” or “no,” without details regarding the timeframe or longevity involved. Similarly, average glucose levels were not recorded, and diabetes severity was not considered as a risk factor. Finally, the major weakness of this study was the lack of detailed information regarding the unintended DTs themselves, such as the location of the dural tear in each case, the size of the dural tear, the operation performed at the time, and the instruments used.

## Conclusion

In this study, based on univariate analysis results from 608 patients, age, revision surgery, LSS, and ULBD were found to be significant risk factors for DT. By understanding these risk factors, surgeons may be better prepared for this kind of complication and should ensure that patients are appropriately informed prior to surgery.

## Data Availability

The datasets used in this study are available from the corresponding author on reasonable request.

## Abbreviations

BETLIF: Biportal endoscopic transforaminal lumbar interbody fusion
BMI: Body mass index
CI: Confidence intervals
CSF: Cerebrospinal fluid
DLS: Degenerative lumbar spondylolisthesis
DT: Dural tear
LDH: Lumbar disc herniation
LSS: Lumbar spinal stenosis
RR: Relative risk
UBE: Unilateral biportal endoscopic
UD: Unilateral decompression
ULBD: Unilateral laminotomy for bilateral decompression
